# A Severe Case of Ipilimumab-Induced Ileocolitis Refractory to Glucocorticosteroids and Infliximab

**DOI:** 10.1155/2018/2431503

**Published:** 2018-02-06

**Authors:** Behdod Poushanchi, Hiren Vallabh, Gorman Joel Reynolds

**Affiliations:** ^1^Department of Medicine, West Virginia University, Morgantown, WV, USA; ^2^Division of Gastroenterology, West Virginia University, Morgantown, WV, USA

## Abstract

Ipilimumab is a monoclonal antibody that works as an immunotherapeutic agent through selective targeting of T cells to strengthen the response to metastatic melanoma. It is well known that this pharmaceutical agent can cause the adverse effect of colitis. We report a rare presentation of ileocolitis refractory to both glucocorticosteroids and infliximab with a resultant pneumatosis and perforation requiring subtotal colectomy and end ileostomy.

## 1. Introduction

The incidence of melanoma has been increasing and currently 132,000 melanoma skin cancer cases occur globally each year [1]. In the United States, it is estimated that 87,110 new cases and 9,730 deaths will result in 2017 alone [2]. Ipilimumab (Yervoy) is an immunotherapeutic agent utilized for the treatment of metastatic melanoma. Ipilimumab works as a cytotoxic T-lymphocyte-associated antigen 4 (CTLA-4) to potentiate the antitumor T-cell response [3]. The most common immune-mediated adverse event associated with ipilimumab is enterocolitis [4]. A case report of ipilimumab-inducing ileocolitis that was refractory to both glucocorticosteroid and infliximab therapy has rarely been presented in the literature.

## 2. Case Report

**Figure 1:**
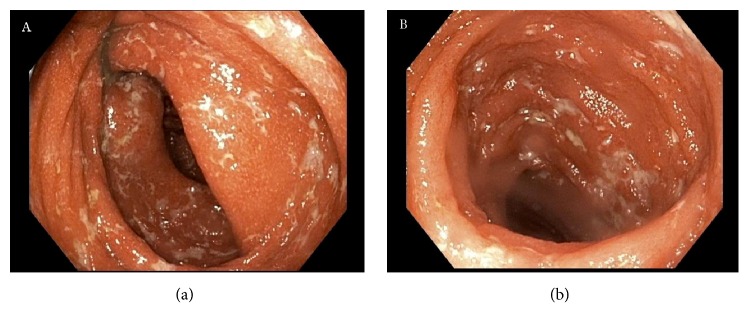
Colonoscopy displaying circumferential, diffuse, edematous, erythematous, and friable mucosa with superficial ulcerations in the (a) colon and (b) terminal ileum.

**Figure 2:**
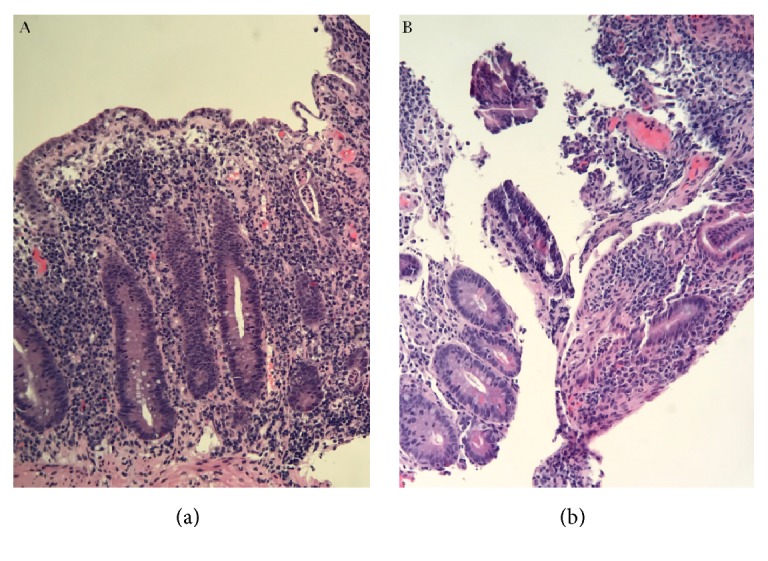
High-power view of chronic active ileocolitis with acute cryptitis, crypt abscess, and granulation tissue in the (a) colon and (b) terminal ileum.

**Figure 3:**
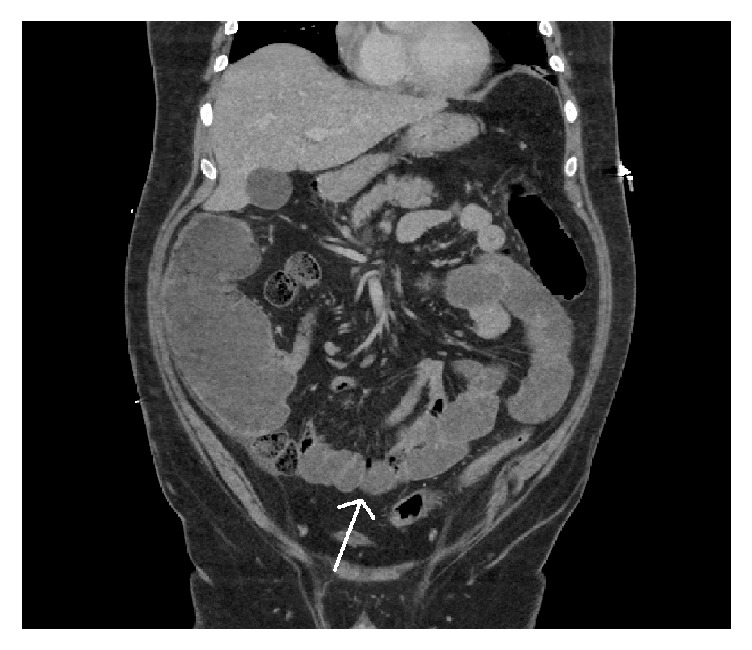
Coronal view from CT enterography demonstrating narrowing of the distal and terminal ileum as well as a short segment of narrowing in the mid ileum (arrow) with fecalization in the distal ileum.

**Figure 4:**
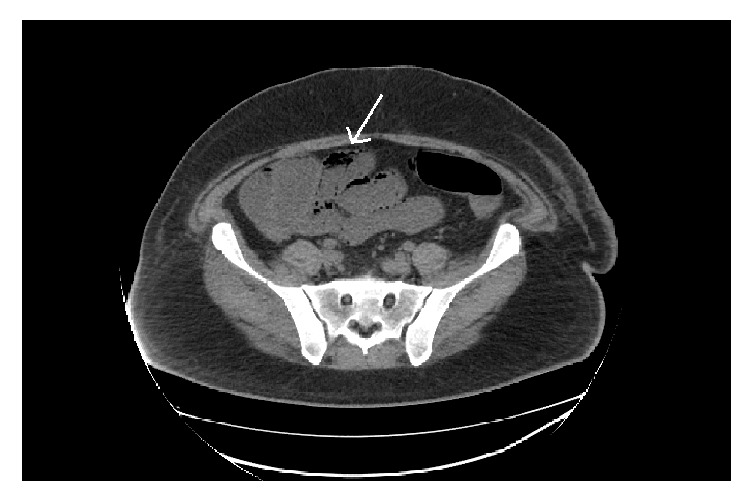
Axial view from CT abdomen exhibiting prominent ascending and transverse colitis with pneumatosis (arrow) of the distal small bowel, perforated abdominal viscus with moderate pneumoperitoneum and pneumomediastinum.

A 39-year-old male presented to our institution with a 3-week history of nausea, vomiting, and diarrhea. Medical history was notable for occipital melanoma excised in 2011. Subsequently during a dental procedure in 2016, the patient was noted to have left sided lymphadenopathy. He underwent a lymph node biopsy, which was positive for metastatic melanoma which resulted in a full neck dissection. Two months thereafter and four weeks prior to his symptom onset, his primary oncologist started him on ipilimumab. The patient then presented to the medical intensive care unit with hypovolemic shock secondary to intractable diarrhea. In addition to fluid resuscitation, patient was continued on his prednisone 50 mg oral twice daily which had been started by his oncologist. Patient's severe diarrhea persisted which prompted endoscopic examination. Colonoscopy showed circumferential, diffuse, edematous, erythematous, and friable mucosa with superficial ulcerations along the entire length of the colon and the terminal ileum (Figures 1(a) and 1(b)). Pathology from the biopsies obtained during the colonoscopy was consistent with chronic active ileocolitis with acute cryptitis, crypt abscess, and granulation tissue (Figures 2(a) and 2(b)). The steroid regimen was consequently changed to intravenous methylprednisolone 20 mg every 8 hours. The patient also underwent a computed tomography enterography which demonstrated narrowing of the distal and terminal ileum as well as a short segment of narrowing in the mid ileum with fecalization in the distal ileum (Figure 3). Additionally, pericolonic stranding was noted to be prominent in the descending colon. Unfortunately, the patient did not respond to intravenous methylprednisolone over the course of the next 8 days and a decision was made to initiate infliximab at 5 mg/kg. Despite the start of infliximab, the patient continued to worsen with diffuse abdominal pain and persistent diarrhea. A repeat computed tomography of the abdomen and pelvis showed prominent ascending and transverse colitis with pneumatosis of the distal small bowel, perforated abdominal viscus with moderate pneumoperitoneum and pneumomediastinum (Figure 4). The patient underwent a subtotal colectomy with end ileostomy. The patient's symptoms improved thereafter and almost a month into the hospital admission, the patient was discharged on an oral methylprednisolone taper.

## 3. Discussion

In a randomized controlled trial, ipilimumab showed significant improvement in overall survival of patients with metastatic melanoma [3]. However, along with the pharmacotherapy well-documented immune-related adverse events have come [4]. In a phase III trial, 60% of patients treated with ipilimumab experienced immune-related adverse events [5]. Esophagitis, gastritis, ileitis, and colitis have all been reported in the literature and given the potential for this pharmacologic agent to deregulate the immune system, an adverse reaction can occur in any organ in the body. With regard to autoimmune colitis, recommendations for initiation of steroids and infliximab for steroid-refractory cases have proven to be life-saving [6]. However, when a case of ileocolitis proves to be refractory to both steroids and infliximab with a resulting perforation, surgical intervention is ultimately pursued. As immunotherapeutic agents become commonplace, it is important for gastroenterologists to remain attentive and keenly observant for complications with an emphasis placed upon early intervention.
